# Genome-wide transcriptomics revealed carbon source-mediated gamma-aminobutyric acid (GABA) production in a probiotic, *Lactiplantibacillus pentosus* 9D3

**DOI:** 10.1016/j.heliyon.2025.e41879

**Published:** 2025-01-10

**Authors:** Nachon Raethong, Pitakthai Chamtim, Roypim Thananusak, Kanyawee Whanmek, Chalat Santivarangkna

**Affiliations:** a Institute of Nutrition, Mahidol University, Nakhon Pathom, 73170, Thailand; b Academic Service Division, National Laboratory Animal Center, Mahidol University, Nakhon Pathom, 73170, Thailand; c Omics Center for Agriculture, Bioresource, Food and Health Kasetsart University (OmiKU), Faculty of Science, Kasetsart University, Bangkok, 10900, Thailand; d Duckweed Holobiont Resource & Research Center (DHbRC), Faculty of Science, Kasetsart University, Bangkok, 10900, Thailand

## Abstract

GABA-producing probiotics present promising opportunities for developing functional foods. Carbon sources have been identified as a critical influence on GABA production. Therefore, this study investigated the holistic metabolic responses and GABA biosynthesis to various carbon sources of *Lactiplantibacillus pentosus* 9D3, a proficient GABA producer, using a genome-wide transcriptomic approach. The analysis revealed 414 genes with differential expression responses to altering carbon sources, i.e., glucose, sucrose, and lactose, notably sugar phosphotransferase systems (PTS) (11 genes), indicating carbon source-mediated transcriptional change patterns in *L. pentosus* 9D3. The integration of transcriptome data with a genome-scale metabolic network (GSMN) revealed that *L. pentosus* 9D3 displays adaptability by synthesizing GABA as an alternative acid-tolerant mechanism when lactose is used as a carbon source rather than depending on the fatty acid synthesis and the arginine catabolic pathway. The findings of this study offer valuable insights into optimal carbon source utilization and gene expression co-regulation, thereby enhancing the GABA-producing capability of a probiotic and broadening its potential applications in the functional food industry.

## Highlights

• The integrative physiology of *L. pentosus* 9D3, a GABA-producing probiotic, was examined, and a transcriptional analysis was performed.
• Carbon sources were found to influence transcriptional changes in acid tolerance and GABA production.
• Lactose activated the GAD operon, increasing GABA production by suppressing other acid tolerance mechanisms such as fatty acid synthesis and arginine catabolism.

## 1 Introduction

Gamma-aminobutyric acid (GABA) is known to have several benefits for physiological functions such as synaptic neurotransmission [1], blood pressure regulation [2], immunity enhancement [3,4], and glucose homeostasis [5]. Moreover, GABA is a functional component in food that is not limited to tranquilizer effects on insomnia [6], depression [6], and autonomic nervous system disorders such as hypertension [7], but also extends to therapeutic effects in preventing and treating non-communicable diseases (NCDs) [8,9]. Given its significant role in promoting health, incorporating GABA has become a key strategy for creating healthier and more nutritious food options.

Lactic acid bacteria (LAB) are essential for food fermentation, with GABA-producing LAB strains offering significant potential for functional food development [[10], [11], [12], [13], [14]]. The enzyme glutamate decarboxylase (GAD) is important for GABA production and helps LAB survive acidic environments by lowering intracellular pH [15]. Optimizing fermentation processes, gene expression, and metabolic pathways to enhance GAD activity is crucial for increasing GABA yields [[16], [17], [18]]. Several factors influence GABA production, including temperature, pH, fermentation time, and nutritional additives [[19], [20], [21]]. Carbon sources have emerged as a factor mediating metabolic adaptations and influencing GABA production [22]. Previous studies reported that the optimal carbon sources for GABA production in various bacteria were glucose, xylose, and galactose [[22], [23], [24], [25]]. Specifically, glucose was identified as the preferable carbon source for high GABA production [22], while xylose was found to improve GABA production in *Levilactobacillus brevis* [23] and *Lentilactobacillus buchneri* [24]. *Lacticaseibacillus rhamnosus* demonstrated high GABA synthesis using galactose as a carbon source [25]. Despite extensive omics-based studies on LAB metabolism [[26], [27], [28], [29]], exploration concerning the influence of different carbon sources on GABA biosynthesis and GAD enzyme activity remains inadequate.

The present study of genome-wide transcriptomics was conducted in *Lactiplantibacillus pentosus* 9D3, which is an efficient GABA producer isolated from Thai fermented food. It has been thoroughly assessed for its safety and probiotic properties [10,30]. This analysis focused on dissecting the holistic metabolic traits of the strain involved in GABA biosynthesis to various carbon sources, including glucose, sucrose, and lactose. The rationale for using glucose, sucrose, and lactose as carbon sources in this study is their widespread involvement in fermentation. Glucose is a universal energy source, preferred in microbial cultures due to its high metabolic efficiency and ability to support rapid growth. Sucrose is an affordable, easily obtainable, and sustainable carbon source that significantly lowers production costs across various industrial sectors. Lactose, primarily present in milk, is crucial for dairy fermentation and the production of fermented products such as yogurt and cheese. Furthermore, the study involved the construction of a genome-scale metabolic network (GSMN) to seamlessly integrate the differentially expressed genes (DEGs) with key metabolites associated with GABA production. This study sheds light on the factors influencing GABA yield in a probiotic, such as carbon source utilization and gene expression patterns, emphasizing the potential impact on functional food development through emerging systems and synthetic biology approaches.

## 2 Materials and methods

### 2.1 Medium and culture conditions

**Fig. 1:**
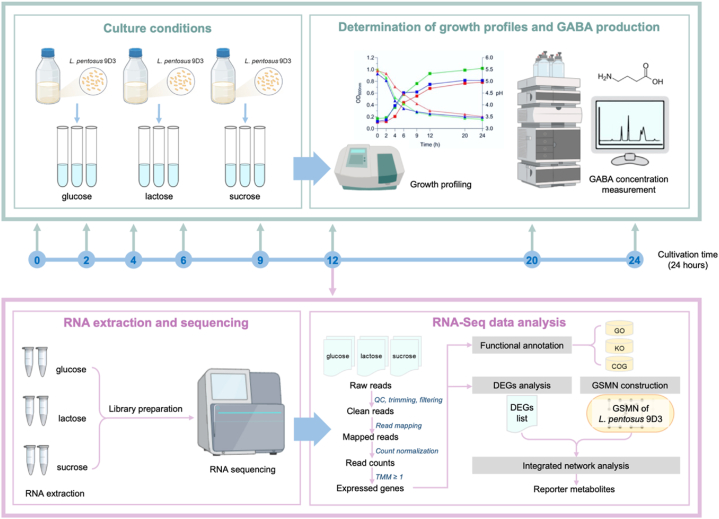
Systematic workflow of genome-wide transcriptomic and genome-scale metabolic network (GSMN)-driven analysis of *L. pentosus* 9D3, a proficient GABA producer.

The study used *L. pentosus* 9D3. The bacterium was grown in an MRS broth at 37 °C for 18–20 h and then transferred to a sterile culture medium with a 2 % (v/v) inoculum of 10^7^ CFU mL^−1^ 9D3. The medium used for cultivation consisted of the following components per liter: yeast extract (5.0 g), K_2_HPO_4_ (2.0 g), (NH_4_)_2_SO_4_ (1.0 g), MgSO_4_·7H_2_O (0.05 g), Tween 80 (1 % (v/v)), and a carbon source at a concentration of 20 g per liter (e.g., glucose, lactose, or sucrose) [29]. Cultivation was performed in triplicate at 37 °C under anaerobic conditions with minimal agitation for 24 h. Cell cultures were sampled at specific times (0, 2, 4, 6, 9, 12, 20, and 24 h) to measure GABA concentrations and for growth profiling, as shown in Fig. 1.

### 2.2 GABA measurement

Culture broth was used to measure the concentration of GABA using dinitrofluorobenzene (DNFB) for derivatization [31,32]. This involved mixing the samples, 1 % DNFB solution, and 0.5M NaHCO_3_ in a 1:1:1 ratio. The mixed sample was incubated at 60 °C for 1 h, followed by dilution with 0.01M KH_2_PO_4_ to adjust the concentration. High-Performance Liquid Chromatography (HPLC) was used for GABA analysis. A combination of acetonitrile and 0.01M KH_2_PO_4_ in a 20:80 ratio was used as a mobile phase with a flow rate of 0.8 mL min^−1^. The analysis was conducted at 35 °C using a 4.6 mm × 250 mm, 5 μm Inertsil ODS-3 C18 column (Shimadzu, Japan). Detection was achieved using a Photo Diode Array detector (SPD-M40) at a wavelength of 360 nm. A standard curve was constructed to determine GABA concentrations. The results were reported as mean ± standard deviation and statistically analyzed using one-way ANOVA and Bonferroni's multiple comparison test, with a significance threshold of *p*-value <0.05. The calculations were performed using GraphPad Prism version 10.1.2.

### 2.3 RNA extraction and sequencing

Cells were harvested at 12h from the two independent cultures grown on three carbon sources for transcriptome analysis: glucose, sucrose, and lactose (Fig. 1). The number of replications used for transcriptome analysis was two replicates, adopted based on a previous study aimed at examining the metabolic responses of a probiotic to various carbon sources [28]. In addition, the decision to collect the cells at 12h was intentional, as this timing ranges with the exponential growth phase. In this phase, bacteria multiply quickly, increasing their responsiveness to changes in carbon sources and facilitating GABA production, thereby providing important insights into metabolic responses. The harvested cells were preserved in RNAprotect tissue reagent (Qiagen, Germany) and kept at −80 °C. Total RNA samples were then extracted from the frozen cells using the RNeasy mini kit (Qiagen, Germany) according to the manufacturer's recommendations. The purity of the extracted RNA was then assessed by gel electrophoresis and measurement of the absorbance ratio at 260 and 280 nm (A_260_/A_280_), with a ratio of 1.8–2.0, indicating pure RNA. For transcriptome profiling, mRNA was isolated using oligo-dT primers and fragmented to be used as templates for cDNA library synthesis. The sequencing was run through an Illumina NovaSeq 6000 system (Macrogen, Korea) with 100-bp paired-end mode (PE100). Raw data can be accessed in the NCBI Sequence Read Archive (SRA) under BioProject No.: PRJNA988285 with the BioSample No.: SAMN36015186, SAMN36016370, SAMN36016371, SAMN36016372, SAMN36016373, and SAMN36016374. Data records in the SRA database are available for access at https://www.ncbi.nlm.nih.gov/sra/?term=PRJNA988285.

### 2.4 Bioinformatic analysis of RNA sequencing data

Read quality was evaluated using FastQC (Galaxy Version 0.74+galaxy0). Adapter and low-quality sequences were trimmed using Trimmomatic (Galaxy Version 0.38.1) [33], with the following parameters: ILLUMINACLIP using Nextera adapter sequence (pair-end) and SLIDINGWINDOW with a minimum mean quality score of 20. The resulting clean reads were aligned and quantified to the *L. pentosus* 9D3 reference coding sequence (CDS) data downloaded from NCBI-SRA BioProject No.: PRJNA739022 (accessed on July 2, 2023) using Salmon quant (Galaxy Version 1.10.1+galaxy0) with default parameters [34,35]. Counts were adjusted for library size by normalizing and scaling using the TMM (trimmed mean of M values) method. Genes with counts greater than or equal to 1 TMM were retained as expressed. The expressed genes were then annotated for functions using various databases and tools. This involved gene ontology (GO) analysis by PANNZER2 [36], KEGG orthology (KO) identification by KofamKOALA [37], and clusters of orthologous gene (COG) assignment by eggNOG-mapper [38,39]. DEGs between different carbon sources, including lactose versus glucose, lactose versus sucrose, and sucrose versus glucose, were identified using DESeq2 (Galaxy Version 2.11.40.8+galaxy0) with strict criteria, comprising an FDR-adjusted *p*-value less than 0.05 and a fold change (FC) of less than −2 (for down-regulated) or greater than 2 (for up-regulated) as a cutoff [40]. Pearson correlation was used for hierarchical clustering analysis with the complete-linkage method by the R base stats package. Pearson correlation was adopted based on a previous study that identified it as one of the top two effective clustering algorithms for gene expression analysis [41]. The clusters of DEGs were visualized using a heat map generated by the R gplots package.

### 2.5 Genome-wide transcriptomics through integrated network analysis

The construction of the genome-scale metabolic network (GSMN) relied on the genome and physiological data of *L. pentosus* 9D3. The GSMN was initially constructed using a protein homology search by mapping the *L. pentosus* 9D3 genome against the gene-protein-reaction (GPR) associations in a collection of genome-scale metabolic models from the MetaNetX database (https://www.metanetx.org/; accessed on July 23, 2023) [42]. Under the default threshold, only GPR-matched *L. pentosus* 9D3 genes were included in the network. Furthermore, GO, COG, and KEGG databases enhanced the GSMN content according to the orthologous genes [[43], [44], [45], [46], [47], [48]]. The reversibility of metabolic reactions and the names of metabolites were curated using MetaCyc [49], MetaNetX [42], and ChEBI [50] databases. Lastly, as *L. pentosus* 9D3 possesses GABA-producing capability, the GPRs associated with GABA synthesis were checked to verify that the network captured the strain physiology. For genome-wide transcriptional analysis, the gene level statistics (*p*-value and FC) of DEGs were integrated with the constructed GSMN in the bi-partite graph format (metabolites-genes pairs) using the R piano package to search for reporter metabolites [51]. Metabolites with a *p*-value less than 0.005 in either the up or down direction are considered reporter metabolites.

## 3 Results

### 3.1 Probiotic growth profiles and GABA production

**Fig. 2:**
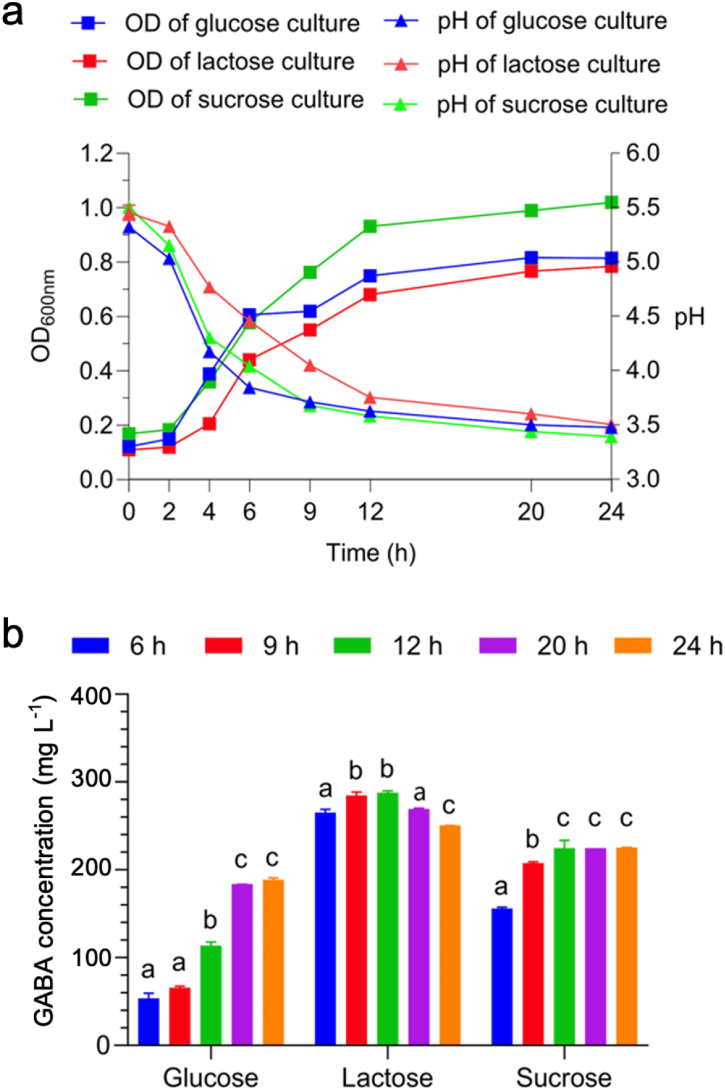
Growth profiles and GABA production of *L. pentosus* 9D3 in various carbon sources. (a) Cell density (OD_600nm_) and pH in glucose, lactose, or sucrose cultures; (b) The measured GABA concentrations in the cultivation broths from glucose, lactose, and sucrose cultures at different times. The letters above the bars represent significant differences at a *p*-value less than 0.05 for each time point within the same carbon source. For example, the GABA concentrations from the glucose cultures at 6h and 9h were not significantly different but differed significantly from those at 12h, 20h, and 24h. Similarly, 20h and 24h were not significantly different but differed significantly from those at 6h, 9h, and 12h. Thus, "a" was assigned to 6h and 9h, "b" to 12h, and "c" to 20h and 24h.

The growth profiles of the *L. pentosus* 9D3 probiotic in glucose, lactose, or sucrose cultures are shown in Fig. 2a. The results indicated that the strain exhibited robust growth, leading to a reduction in pH as a result of lactic acid production across all carbon sources. Among these, glucose was favorable for supporting 9D3 growth, with a significant enhancement observed compared to lactose and sucrose cultures, as indicated by a higher maximum specific growth rate (μ_max_) of 0.114 ± 0.003 per hour (h^−1^) (*p*-value = 0.004) (Table 1). The pH dropped to 3.85 ± 0.03 within 6 h in the glucose culture, which was nearly twice as fast as in the lactose culture. However, once the GABA content was considered, the lactose culture offered remarkably different GABA production than glucose and sucrose cultures, as shown in Fig. 2b. In addition, the GABA contents showed notable differences among the various time points within the same carbon source (Fig. 2b). In the glucose culture, GABA concentration increased significantly from early (times: 6h and 9h) to late (times: 20h and 24h) fermentation and showed the highest value (188.58 ± 1.59 mg L^−1^) at 24 h. Likewise, the GABA concentration in the sucrose culture at 12 h increased significantly compared to the initial concentration at 6 h and remained constant until late (times: 20h and 24h) fermentation. Considering the highest GABA production found in the lactose culture, GABA concentration accumulated rapidly during the exponential growth phase and reached the peak value (287.37 ± 1.69 mg L^−1^) at 12 h, which was significantly higher by 1.5 and 1.3 fold (*p*-value <0.0001) compared to the highest GABA values obtained from glucose and sucrose cultures, respectively. Thereafter, the GABA concentration decreased slightly to 250.39 ± 0.09 mg L^−1^ at 24 h in the lactose culture while being significantly higher than both carbon sources (*p*-value <0.0001).

**Table 1:** Effects of glucose, sucrose, and lactose on the maximum specific growth rate and maximum GABA production of *L. pentosus* 9D3; data are presented as means ± SD (n = 3).

Parameters	Glucose	Sucrose	Lactose	*p*-value
Maximum specific growth rate: μ_max_ (h^−1^)	0.114 ± 0.003^a^	0.099 ± 0.0003^a^	0.058 ± 0.0001^b^	0.004
Maximum GABA production (mg L^−1^) a	188.58 ± 1.59^a^	225.15 ± 0.23^b^	287.37 ± 1.69^c^	<0.0001

a The maximum GABA production values were observed at times of 24, 24, and 12 h for glucose, sucrose, and lactose cultures, respectively. Super subscripts indicate significant differences between the studied groups at *p*-value <0.05.

### 3.2 Transcriptome data summary of *L. pentosus* 9D3

**Fig. 3:**
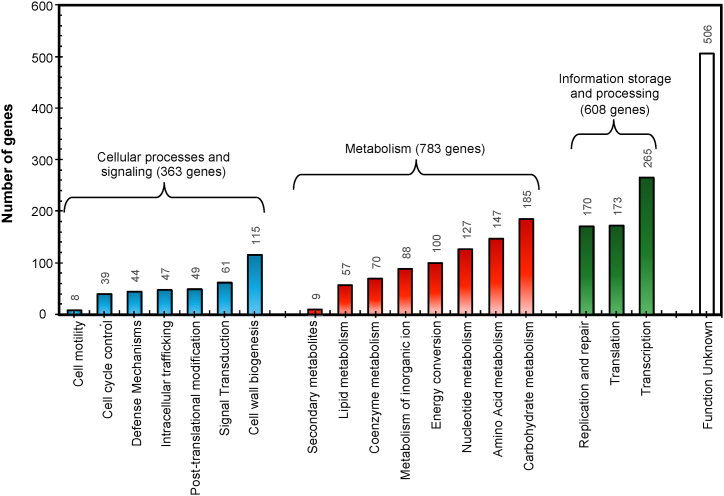
Distribution of expressed genes across COG functional categories.

The RNA-sequencing (RNA-seq) libraries of *L. pentosus* 9D3 cultivated in glucose, sucrose, and lactose resulted in a total of 18.42 billion base pairs of raw reads. Following adapter and quality trimming, the average total clean reads amounted to 30.77 ± 2.43, 33.17 ± 0.31, and 27.25 ± 4.28 million reads for glucose, sucrose, and lactose cultures, respectively (Supplementary Table S1). The clean reads for each library were mapped to the reference *L. pentosus* 9D3 genome, revealing 2692 expressed genes from all cultures. Of these, 2429 expressed genes were predicted protein functions regarding COGs (2260 genes), GO (2239 genes), and KO (1530 genes) databases. The COG database analysis identified 19 functional categories across 2260 genes, with the most predominant metabolic functions including the metabolism of carbohydrates, amino acids, nucleotides, energy conversion, inorganic ion transport, coenzymes, lipids, and secondary metabolites (Fig. 3). The findings from the COG analysis were consistent with the results from KEGG identifiers, indicating that metabolism accounted for the majority of gene functions, with 803 out of 1530 genes associated with metabolic functions (Supplementary Table S2).

### 3.3 DEG analysis to compare gene expression in different carbon sources

**Fig. 4:**
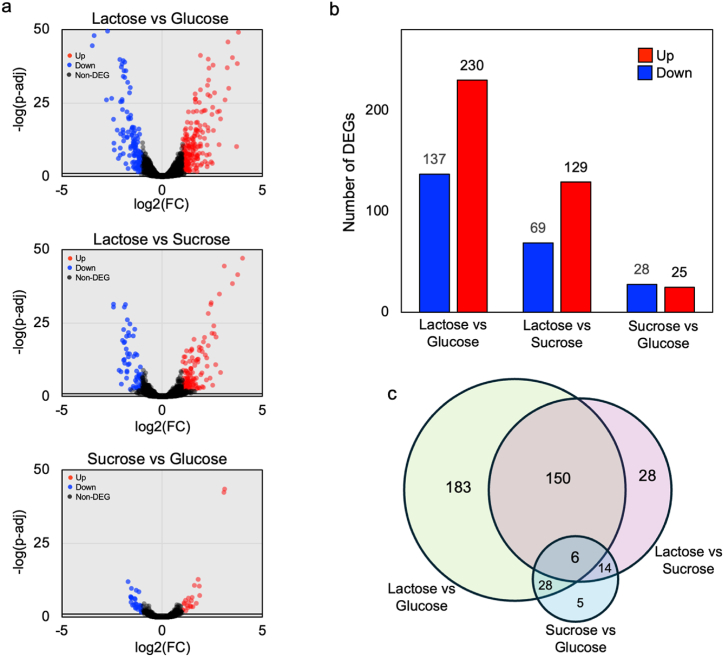
DEG analysis for comparing gene expression in different carbon sources. (a) Volcano plots show the DEGs in each comparison. The significantly up-regulated and down-regulated expressed genes are represented by red and blue dots, respectively. The black dots do not indicate significant differentially expressed genes.; (b) The number of DEGs in each comparison is shown in the bar chart.; (c) The Venn diagram illustrates non-redundant DEGs across all comparisons. The datasets used for DEG analysis included SAMN36015186 and SAMN36016370 for the glucose culture, SAMN36016373 and SAMN36016374 for the sucrose culture, and SAMN36016371 and SAMN36016372 for the lactose culture.

The transcriptome data involved three comparisons: sucrose versus glucose, lactose versus sucrose, and lactose versus glucose (Fig. 4a and b). The numbers of DEGs were 53 for sucrose versus glucose, 198 for lactose versus sucrose, and 367 for lactose versus glucose, totaling 414 non-redundant DEGs (Fig. 4c and Supplementary Table S3). Comparisons revealed significantly higher numbers of DEGs for lactose versus sucrose and lactose versus glucose compared to sucrose versus glucose.

When examining individual carbon sources, it was observed that, in the glucose culture, the DEGs encoding enzymes for the arginine metabolism such as acetyl-gamma-glutamyl-phosphate reductase (KSF55_02445), acetylglutamate kinase (KSF55_02455), acetylornithine aminotransferase (KSF55_02460), glutamate acetyltransferase (KSF55_02450), ornithine carbamoyltransferase (KSF55_02465), arginosuccinate lyase (KSF55_03925), and arginosuccinate synthase (KSF55_03920) exhibited significantly higher expression levels in glucose cultures than in those grown on sucrose or lactose. Furthermore, among the genes related to sugar phosphotransferase systems (PTS), 11 genes were differentially expressed depending on the carbon source used, indicating carbon source-mediated transcriptional change patterns in *L. pentosus* 9D3, as listed in Table 2. Out of these, 5 genes, such as mannose PTS, mannitol PTS, and acetylglucosamine PTS, were up-regulated, while 6 genes, such as alpha-glucoside PTS and galactitol PTS, were down-regulated in glucose cultures compared to sucrose or lactose cultures. In the sucrose culture, the DEGs related to glycerophospholipid metabolism, including alpha-glycerophosphate oxidase (KSF55_01840) and glycerol kinase (SF55_01835), were up-regulated compared to the glucose culture. Additionally, 6-phosphogluconate dehydrogenase (KSF55_05970) presented a higher expression level in the cells grown on sucrose compared to glucose. When analyzing lactose cultures, 4 DEGs associated with galactose metabolism, such as beta-galactosidase (KSF55_15450 and KSF55_15455) and galactokinase (KSF55_15460), were up-regulated relative to glucose and sucrose cultures. Moreover, the expression level of the KSF55_00870, which encodes arabinogalactan oligomer/maltooligosaccharide transport system substrate-binding protein, was very low in cultures using sucrose and glucose but showed significant upregulation in lactose cultures.

**Table 2:** List of DEGs related to sugar phosphotransferase systems (PTS).

Gene ID	Protein function	Glucose^a^	Sucrose^b^	Lactose^c^
KSF55_03040	Mannose PTS	Up		Down
KSF55_03010	Mannose PTS	Up	Down	Down
KSF55_01155	Mannitol PTS	Up	Down	Down
KSF55_01145	Mannitol PTS	Up		Down
KSF55_11445	Acetylglucosamine PTS	Up		Down
KSF55_16135	Alpha-glucoside PTS	Down		Up
KSF55_13515	Cellobiose PTS	Down		Up
KSF55_15895	Galactitol PTS	Down		Up
KSF55_15890	Galactitol PTS	Down		Up
KSF55_15680	Ascorbate PTS	Down	–	Up
KSF55_00925	Sucrose PTS	Down	Up	–

a The up- or down-regulated DEGs in the glucose culture were considered by comparing glucose to sucrose or lactose cultures.

b The up- or down-regulated DEGs in the sucrose culture were considered by comparing sucrose to glucose cultures.

c The up- or down-regulated DEGs in the lactose culture were considered by comparing lactose to glucose cultures.

**Fig. 5:**
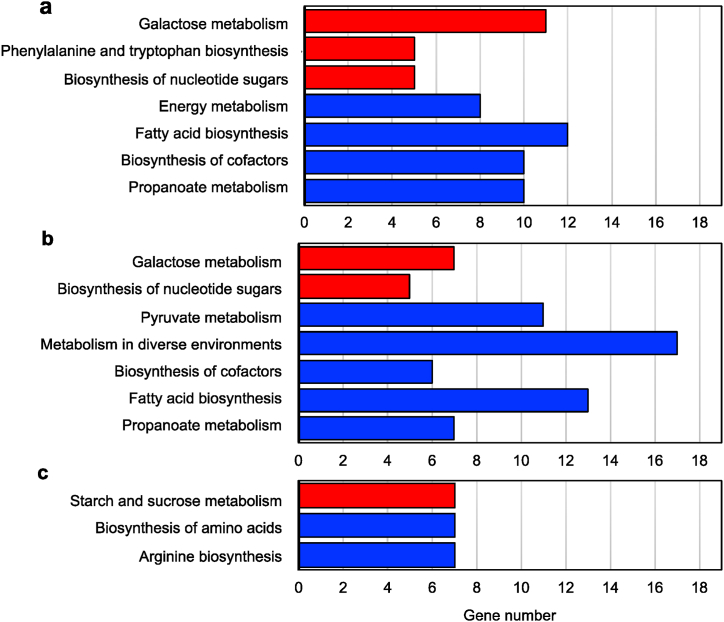
KEGG pathway enrichment analysis of DEGs showing (a) lactose versus glucose, (b) lactose versus sucrose, and (c) sucrose versus glucose. Enriched KEGG pathways are highlighted in red (up-regulated DEGs) and blue bars (down-regulated DEGs) based on a *p*-value threshold of <0.01.

The DEGs associated with KEGG identifiers were examined through pathway enrichment analysis, revealing the regulatory mechanisms governing metabolic processes in *L. pentosus* 9D3 response to variations in carbon sources, as depicted in Fig. 5. Upon comparing the cultures of lactose versus glucose (Fig. 5a) and lactose versus sucrose (Fig. 5b), the up-regulated DEGs in the metabolism of galactose and nucleotide sugars were observed to be more significantly enriched in the lactose culture, such as KSF55_15450, KSF55_15440, and KSF55_04140, which encode beta-galactosidase (EC: 3.2.1.23), UDP-glucose 4-epimerase (EC: 5.1.3.2) and glutamine-fructose-6-phosphate transaminase (EC: 2.6.1.16), respectively.

In contrast, most down-regulated genes in the lactose culture were associated with propanoate metabolism, biosynthesis of cofactors, and fatty acid biosynthesis. Comparing sucrose and glucose (Fig. 5c), the up-regulated DEGs in starch and sucrose metabolism were more enriched in the sucrose culture, such as KSF55_00920, KSF55_00925, KSF55_00930, and KSF55_00940, which encode fructokinase, sucrose PTS system, beta-fructofuranosidase, and oligo-1,6-glucosidase, respectively.

### 3.4 Carbon source-mediated transcriptional change patterns in *L. pentosus* 9D3

**Fig. 6:**
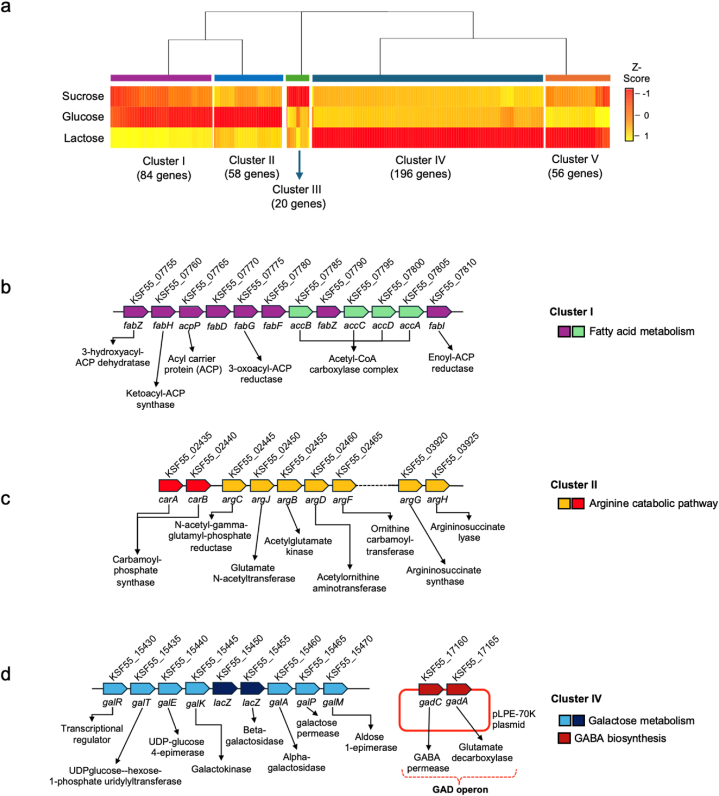
Carbon source-mediated transcriptional change patterns and syntenic gene sets of DEGs. Additional abbreviations and functions of gene names are as follows: *fabD*; acyl carrier protein (ACP) S-malonyltransferase, and *fabF*; beta-ketoacyl-ACP synthase.

A hierarchical clustering analysis compared the gene expression patterns of three carbon sources: lactose, sucrose, and glucose. After examining the correlation in transcriptional change patterns of DEGs, five clusters were identified (Fig. 6 and Supplementary Table S4).

Cluster I showed the up-regulated transcriptional patterns of a syntenic gene set (SGS) associated with fatty acid metabolism, such as acetyl-CoA carboxylase complex, acyl carrier protein (ACP), ketoacyl-ACP synthase, 3-hydroxyacyl-ACP dehydratase, enoyl-ACP reductase, and 3-oxoacyl-ACP reductase) when either sucrose or glucose was used as a carbon source (Fig. 6a and b). In contrast, Cluster II showed the utilization of glucose led to up-regulated transcriptional patterns in an SGS responsible for the arginine catabolic pathway (Fig. 6a and c). This involved carbamoyl-phosphate synthase (KSF55_02435 and KSF55_02440), ornithine carbamoyltransferase (KSF55_02465), arginosuccinate synthase (KSF55_03920), and arginosuccinate lyase (KSF55_03925). Furthermore, the presence of lactose or sucrose resulted in a significant decrease in the expression patterns of specific genes in the mannose and mannitol PTS, such as KSF55_03010, KSF55_03040, KSF55_01145, and KSF55_01155, indicating potential co-regulation by the glucose signaling of genes in Cluster II.

In the lactose culture, the SGS encoding enzyme involved in galactose metabolism, including UDPglucose-hexose-1-phosphate uridylyl transferase (KSF55_15435), UDP-glucose 4-epimerase (KSF55_15440), galactokinase (KSF55_15445), beta-galactosidase (KSF55_15450 and KSF55_15455), and galactose permease (KSF55_15465), showed up-regulated transcriptional patterns in Cluster IV (Fig. 6a and d). Additionally, Cluster IV was enriched with many genes encoding enzymes involved in sugar alcohol utilization, such as L-iditol 2-dehydrogenase (KSF55_15875 and KSF55_15880) and galactitol PTS (KSF55_15890, and KSF55_15895). The up-regulated transcriptional patterns of these genes were similar to the ubiquitous expression of catabolite control protein A (CcpA) encoded by KSF55_10355, which was activated when lactose was used as a carbon source. Moreover, many up-regulated genes during the CcpA activation were located on prophage (23 genes) and plasmids (16 genes). These also included the up-regulation of glutamate decarboxylase (GAD) operon, i.e., KSF55_17160 (*gadC*) and KSF55_17165 (*gadA*), located on pLPE-70K plasmid. The findings indicate that lactose, as an alternative carbon source, affects not only the transcriptional changes in the central carbon metabolism but also activates the expression of accessory genes encoded by prophages and plasmids, notably GAD operon, in *L. pentosus* 9D3.

### 3.5 Genome-wide transcriptomics to dissect holistic metabolic responses associated with GABA production in *L. pentosus* 9D3

A GSMN of *L. pentosus* 9D3 was constructed, consisting of 734 genes, 708 metabolites, and 1364 metabolic reactions (Supplementary File 1). This GSMN was utilized to examine the holistic metabolic changes in response to a shift in carbon sources. To achieve this, a genome-wide transcriptomic analysis was applied to identify the reporter metabolites. In response to lactose compared to glucose cultures, nine reporter metabolites were affected by transcriptional up-regulation, as listed in Table 3. Interestingly, the presence of lactose in the culture medium caused an increase in the activity of genes encoding prephenate dehydratase (KSF55_09285), chorismate synthase (KSF55_09280), and chorismate mutase (KSF55_05230). These genes play a significant role in the biosynthesis of aromatic amino acid intermediates such as 3-enolpyruvyl-shikimate 5-phosphate, prephenate, and chorismate. Additionally, the transcriptional up-regulation of GABA biosynthetic genes, including GABA aminotransaminase (KSF55_08005) and GAD operon (KSF55_17160 and KSF55_17165), was observed in response to the lactose culture. Compared to GABA biosynthesis, 60 reporter metabolites were impacted by transcriptional down-regulation when exposed to the lactose culture. These reporter metabolites primarily influenced fatty acid metabolism, including long-chain unsaturated fatty acids such as hexadecenoate, tetradecanoate, myristoyl-ACP, and dodecanoyl-ACP (Table 4). This result aligns with the down-regulation of genes involved in fatty acid metabolism, which are highly active in the presence of glucose. Additionally, the production of arginosuccinate, a crucial precursor in arginine metabolism, was specifically suppressed in the lactose culture.

**Table 3:** List of reporter metabolites affected by transcriptional up-regulation in response to lactose compared to glucose cultures.

Reporter metabolites	Up directional *p*-value
Alpha-D-galactose 1-phosphate	0.001996
Prephenate	0.001996
UDP-alpha-D-galactose	0.003992
Galactitol 1-phosphate	0.003992
Alpha-D-galactose	0.003992
3-enolpyruvyl-shikimate 5-phosphate	0.003992
Chorismate	0.003992
3-methylbutanoyl-CoA	0.003992
Gamma-aminobutyric acid (GABA)	0.003992

**Table 4:** List of 10 selected reporter metabolites affected by transcriptional down-regulation in response to lactose compared to glucose cultures.

Reporter metabolites	Down directional *p*-value
Coenzyme A (CoA)	0.001996
Acetyl-CoA	0.001996
Malonyl-CoA	0.001996
Methylmalonyl-CoA	0.001996
Long-chain unsaturated fatty acid (e.g., hexadecenoate)	0.001996
Tetradecanoate	0.001996
Myristoyl-acyl carrier protein (ACP)	0.001996
Dodecanoyl-ACP	0.001996
Arginosuccinate	0.001996
NADPH	0.003992

## 4 Discussion

*L. pentosus* 9D3 is a safe LAB strain that produces GABA and can potentially be used as a probiotic [10,30]. Optimizing the GABA-producing capability of *L. pentosus* 9D3 is critical for market demands. According to physiological studies aimed at maximizing the growth and GABA production of *L. pentosus* 9D3, the results indicate that glucose is an inducer for fast growth and acidification, while lactose is favorable for GABA production (Fig. 2).

Identification of significant genes from DEGs analysis and reporter metabolites revealed the metabolic responses of *L. pentosus* 9D3 to carbon source alteration. In glucose- or sucrose-rich media, the activation of genes involved in producing acetyl-CoA and fatty acids was observed (Fig. 6a and b). This result is consistent with previous reports on LAB, including *Lactobacillus casei*, *Streptococcus gordonii*, and *Streptococcus salivarius*, which were found to alleviate cell damage caused by acidic environments by increasing the production of long-chained and mono-unsaturated fatty acids [52]. Increased fatty acids are associated with the ability of LAB to maintain membrane integrity and fluidity when exposed to acid stress, such as an extremely acidic stomach, which is relevant to probiotic properties [53,54]. Our previous study revealed that *L. pentosus* 9D3 has good survivability in hostile stomach conditions, i.e., pH 2.0 [30]. This suggests that *L. pentosus* 9D3 may stimulate fatty acid synthesis to increase long-chain unsaturated fatty acid (e.g., hexadecenoate) and consequently improve the fluidity of the cell membrane to counteract environmental stress, particularly in low pH conditions.

In addition, the up-regulation of specific genes involved in arginine-urea metabolism, such as carbamoyl-phosphate synthase, ornithine carbamoyl transferase, arginosuccinate synthase, and arginosuccinate lyase was observed when glucose was used as a carbon source (Fig. 6a and c). Elucidating the amino acid metabolism patterns is also important for optimizing GABA-producing capability. Generally, LAB is a fastidious microorganism that requires amino acids for protein synthesis, precursor biosynthesis, energy generation, pH control, and stress resistance [55,56]. Previous studies showed that arginine is crucial in protecting LAB against damage caused by a low-pH environment and contributing to adaptation to osmotic stress [[57], [58], [59]]. The catabolic pathway of arginine involved in the urea cycle produces NH_3_ and CO_2_, which are essential for pH homeostasis, thus protecting cells against acid stress [60]. These findings indicate that *L. pentosus* 9D3 can cope with acid stress during growth in the glucose culture by co-regulating the transcription of fatty acid synthesis and arginine catabolic pathway.

The down-regulation of genes related to the biosynthesis of acetyl-CoA and arginosuccinate, however, which are precursors toward fatty acid synthesis and arginine catabolism, respectively, was observed upon the alteration of glucose to lactose cultures (Table 4). This may have been due to the catabolite control protein A (CcpA) activation when lactose was used as an alternative carbon source. Carbon catabolite repression (CCR) is a crucial regulatory mechanism in gram-negative and gram-positive bacteria [61]. This mechanism ensures optimal use of available nutrients by regulating bacteria to prioritize using preferred carbon sources when alternatives are available. CcpA, a member of the LacI/GalR family of transcriptional regulators, is a crucial regulator of the CCR mechanism and is highly conserved among gram-positive bacteria [62]. Transcriptomic and physiological studies conducted on *Lactobacillus plantarum* ST-III and its *ccpA* mutant strain have shown that CcpA plays a vital role in regulating not only carbohydrate metabolism but also other cellular processes such as pyruvate metabolism and fatty acid synthesis [63]. In addition, although the arginine catabolic pathway is positively regulated by urea and a low pH [48,52], the metabolic flux toward the arginine-urea cycle was identified as co-regulated by CcpA [64]. This agrees with the decreased expression of specific genes related to the arginine-urea cycle during CcpA activation, implying a complex transcriptional regulatory network of *L. pentosus* 9D3 in response to environmental acidification.

In contrast to fatty acid synthesis and arginine catabolism, the up-regulation of specific genes involved in GABA production, such as GAD operon, was identified when lactose was utilized as an alternative carbon source (Table 3). The activity of the GAD enzyme is one of the key mechanisms crucial to maintaining pH homeostasis in LAB [15]. This mechanism efficiently reduces the proton concentration in bacterial cells by decarboxylating glutamate to produce GABA and then exporting GABA to the extracellular environment. Besides the activation of the GAD enzyme at a low pH [[65], [66], [67]], the results of this study show that using lactose as an alternative carbon source significantly increases the production of GABA and the expression of plasmid-encoding GAD operon (Fig. 2, Fig. 6d).

In summary, the present study revealed the transcriptomic responses of *L. pentosus* 9D3 to glucose, sucrose, and lactose by regulating metabolism, which enhances growth, acid tolerance, and GABA production. It is important to acknowledge a limitation inherent in the number of replicates used for transcriptome analysis, which may heighten the risk of false positive results [68]. To mitigate this, we implemented rigorous statistical analyses in conjunction with GSMN-driven analysis, revealing good agreement in responsive genes and reporter metabolites across the different carbon sources. Additionally, the biological significance of the results was carefully considered, corroborating with existing literature to warrant the findings. Moreover, the study findings indicated that a semi-defined medium with lactose at 37 °C under anaerobic conditions effectively supports probiotic growth and GABA production. Notably, other carbon sources may affect growth and GABA yield, indicating the need for future studies on the metabolic flexibility and GABA production efficiency of the strain on a wider range of carbon substrates. In addition, this study identified metabolites as reporter metabolites, highlighting carbon source-mediated transcriptional changes resulting from complex regulatory mechanisms coordinating different metabolic routes [69]. In a broader view, changes in metabolite concentrations could indicate metabolic responses to the distribution of metabolic flux in central metabolism. Further exploration of integrating omics with metabolic flux analysis has the potential to provide a quantitative and comprehensive depiction of metabolic pathways, as well as to validate the identified reporter metabolites [70].

## 5 Conclusions

*L. pentosus* 9D3 is a safe probiotic strain recognized for its GABA-producing capability. The integration of genome-wide transcriptomics with GSMN-driven analysis has provided valuable insights into the metabolic responses of the strain to various carbon sources. The analysis revealed increased gene expression in acetyl-CoA and fatty acid production as well as arginine-urea metabolism, facilitating adaptation to acid stress in glucose or sucrose cultures. Conversely, the lactose culture down-regulated fatty acid and arginine biosynthesis, possibly due to CcpA-mediated carbon catabolite repression, while enhancing GABA-related gene expression, particularly the GAD operon, to enhance GABA synthesis for pH balance. The study demonstrated metabolic control during the adaptation of *L. pentosus* 9D3 to different carbon sources, specifically glucose, sucrose, and lactose, through transcriptional co-regulation, supporting cell growth, and acid-tolerant mechanisms, particularly GABA synthesis. These insights provide a valuable perspective on optimizing fermentation conditions by utilizing either genetic or physiological methods to improve the properties of the GABA-producing probiotic.

## CRediT authorship contribution statement

**Nachon Raethong:** Writing – review & editing, Writing – original draft, Visualization, Validation, Supervision, Project administration, Methodology, Investigation, Funding acquisition, Data curation, Conceptualization. **Pitakthai Chamtim:** Visualization, Validation, Methodology, Investigation, Formal analysis, Data curation, Conceptualization. **Roypim Thananusak:** Formal analysis, Data curation. **Kanyawee Whanmek:** Resources, Methodology, Investigation, Formal analysis. **Chalat Santivarangkna:** Supervision, Resources, Project administration, Conceptualization.

## Data availability statement

Raw data can be accessed in the NCBI Sequence Read Archive (SRA) under BioProject No.: PRJNA988285 with the BioSample No.: SAMN36015186, SAMN36016370, SAMN36016371, SAMN36016372, SAMN36016373, and SAMN36016374. The dataset associated with BioProject PRJNA988285 is available for access at https://www.ncbi.nlm.nih.gov/sra/?term=PRJNA988285.

## Ethical approval statement

“Not applicable”.

## Funding

This research project is supported by 10.13039/501100004156Mahidol University (grant number: A15/2565).

## Declaration of competing interest

The authors declare that they have no known competing financial interests or personal relationships that could have appeared to influence the work reported in this paper.
